# Discovery of a Novel Retrovirus Sequence in an Australian Native Rodent (*Melomys burtoni*): A Putative Link between Gibbon Ape Leukemia Virus and Koala Retrovirus

**DOI:** 10.1371/journal.pone.0106954

**Published:** 2014-09-24

**Authors:** Greg Simmons, Daniel Clarke, Jeff McKee, Paul Young, Joanne Meers

**Affiliations:** 1 School of Veterinary Science, The University of Queensland, Gatton, Queensland, Australia; 2 Australian Infectious Diseases Research Centre, School of Chemistry and Molecular Biosciences, The University of Queensland, St Lucia, Queensland, Australia; 3 Ecosure, West Burleigh, Queensland, Australia; University of Illinois at Urbana-Champaign, United States of America

## Abstract

Gibbon ape leukaemia virus (GALV) and koala retrovirus (KoRV) share a remarkably close sequence identity despite the fact that they occur in distantly related mammals on different continents. It has previously been suggested that infection of their respective hosts may have occurred as a result of a species jump from another, as yet unidentified vertebrate host. To investigate possible sources of these retroviruses in the Australian context, DNA samples were obtained from 42 vertebrate species and screened using PCR in order to detect proviral sequences closely related to KoRV and GALV. Four proviral partial sequences totalling 2880 bases which share a strong similarity with KoRV and GALV were detected in DNA from a native Australian rodent, the grassland melomys, *Melomys burtoni*. We have designated this novel gammaretrovirus *Melomys burtoni* retrovirus (MbRV). The concatenated nucleotide sequence of MbRV shares 93% identity with the corresponding sequence from GALV-SEATO and 83% identity with KoRV. The geographic ranges of the grassland melomys and of the koala partially overlap. Thus a species jump by MbRV from melomys to koalas is conceivable. However the genus *Melomys* does not occur in mainland South East Asia and so it appears most likely that another as yet unidentified host was the source of GALV.

## Introduction

Koala populations in northern and central eastern Australia are currently undergoing significant declines. Habitat loss, drought, predation and disease have all been incriminated as contributing to these declines [1]–[3] with the main diseases being chlamydiosis, other opportunistic infections and lymphoid neoplasia [4]–[5]. Through possible immunosuppressive and oncogenic mechanisms, koala retrovirus (KoRV) is a potential predisposing factor in the development of these infectious and neoplastic diseases [6]–[8]. Retroviral particles were first observed in koala lymphosarcoma tissue [9] and partial retroviral sequences were detected in koalas with opportunistic infections [10] and in apparently healthy animals [11]. KoRV was subsequently identified in koalas in Queensland, Australia, and fully sequenced and characterised as an intact gammaretrovirus [12]. The virus has since been detected in all captive colonies tested in Australia, and the majority of free-living koala populations [6], [12]–[13], as well as captive animals in the USA, Japan and Germany [10], [14]–[15].

KoRV has attracted considerable scientific interest as a result of its unusual biological and epidemiological features. It is endogenous in northern Australian koala populations, as evidenced by 100% proviral prevalence [13] and the demonstration of KoRV provirus in sperm cells and inherited proviral insertion patterns [7]. However, KoRV also displays features of an exogenous virus, in that individual animals have distinct proviral insertion patterns [7], the KoRV provirus is full-length and transcriptionally-active, plasma from all provirus-positive koalas tested to date has been positive for viral RNA and not all koalas are KoRV positive [6], [12]–[13].

The endogenous/exogenous duality of KoRV has been further highlighted by two recent studies, which found different genetic variants of KoRV in captive koalas in Japan and the US. In both studies the original KoRV, first identified in Australian koalas and referred to by these groups as KoRV-A, was identified in all PCR-positive animals. This retrovirus had previously been shown to use the phosphate transporter Pit-1 as a cellular receptor [16]. In addition, both studies showed that some of these KoRV-A positive animals were also infected with different, apparently exogenous viruses (referred to as KoRV-B in the US or KoRV-J in the Japanese studies), which have significant variations in their respective *env* sequences, leading to the utilization of a different cellular receptor, the thiamine transporter THTR1 [17]–[18].

Some koala populations in southern Australia are either free of the virus or have mixed KoRV proviral prevalence, indicating that the virus is not endogenous in these populations [7], [13]. Thus it appears that KoRV is an active exogenous retrovirus currently undergoing a natural process of endogenisation [7].

A further unusual feature of KoRV is the close genetic relationship it shares with gibbon ape leukemia virus (GALV), an exogenous virus initially isolated from captive white-handed gibbons (*Hylobates lar*) in Thailand with malignant lymphoma and leukemia, and later isolated from other gibbons with lymphoid tumours, gibbons inoculated with human material or as a human cell culture contaminant [19]–[22]. KoRV and GALV share a high degree of homology across the entire viral genome and both viruses form a clade with eutherian (porcine, murine, feline and chiropteran) gammaretroviruses [11], [23]. This phylogenetic relationship and the pathogenicity of the two viruses suggest that neither KoRV or GALV are recombinants nor co-evolved viruses but rather that they have transferred into their respective hosts via “host jumping” events and thus demonstrate a typical pattern of infection seen when a naive host meets a new pathogen [24]–[25].

The “host-jumping” events that produced KoRV and GALV may have involved cross-species transmission between gibbons and koalas or transmission to both species from a common host that is a reservoir for an ancestral virus. Because the geographic ranges of koalas in Australia and gibbons in South East Asia do not overlap [1], [26], a direct species jump appears improbable and we reasoned that transmission from an as yet unidentified intermediate host into both species was more likely. The aims of this work were to identify this intermediate host and discover the putative KoRV-GALV ancestor virus. We screened a total of 42 native or introduced vertebrate species in Australia for the presence of related viruses, including 19 rodent and seven bat species. We considered these taxa to be the most likely candidate species, either because they transit between Australia and South East Asia or their distributions overlap the ranges of both gibbons and koalas and/or their feeding ecology could result in close contact with gibbons and koalas.

## Materials and Methods

### Ethics statement

All animals were treated in accordance with the Australian code for the care and use of animals for scientific purposes. The University of Queensland native and exotic wildlife and marine mammals animal ethics committee approved the active collection of samples assayed in this study (MICRO/PARA/153/06/ARC, SAS/125/09/CRC). All archival samples received from other researchers were approved by the ethics committees of the respective institutions. All live *Melomys burtoni* were trapped under Queensland Department of Environment and Resource Management permit WISP05200108.

### Sample collection

Blood, tissue or DNA samples of 42 animal species were obtained from a variety of sources, including collaborating research groups and wildlife hospitals. Both native species and those introduced to Australia were included in the study (Table 1).

**Table 1:** Species tested using PCR for the presence of koala retrovirus related sequence.

Rodents	Common name	Scientific name	No tested	Sample
	Canefield rat	Rattus sordidus*	3	Skin
	Water rat	*Hydromys chrysogaster*	1	Skin
	Grassland melomys	Melomys burtoni*	17	Skin, spleen, heart, DNA
	Rainforest melomys	*Melomys cervinipes*	13	Skin, spleen, heart, DNA
	House mouse	Mus musculus*	30	Spleen, liver
	Black rat	*Rattus rattus*	5	Spleen, liver
	Bush rat	*Rattus fuscipes*	4	Spleen
	White tailed rat	Uromys caudimaculatus*	3	Heart
	Indochinese forest rat	*Rattus andamanensis*	1	Skin
	Yellow bellied country rat	*Rattus losea*	2	Skin
	Pale field rat	*Rattus tunneyi*	2	Skin
	Small white toothed rat	*Berylmys berdmorei*	2	Skin
	Greater bandicoot rat	*Bandicota indica*	2	Skin
	Savile’s bandicoot rat	*Bandicota savilei*	2	Skin
	Swamp rat	*Rattus lutreolus*	1	Skin
	Lesser bandicoot rat	*Bandicota bengalensis*	2	Skin
	Norway rat	*Rattus norvegicus*	1	Skin
	Cape York rat	*Rattus leucopus*	1	Heart
	Eastern Chestnut mouse	*Pseudomys gracilicaudimaculatus*	1	Skin
**Flying vertebrates**				
	Large flying fox	*Pteropus vampyrus*	4	Skin
	Black flying fox	Pteropus alecto*	28	Spleen, blood
	Spectacled flying fox	*Pteropus conspiculatus*	8	Skin
	Big eared flying fox	*Pteropus macrotis*	1	Skin
	Grey headed flying fox	*Pteropus poliocephalus*	1	Skin
	Little red flying fox	*Pteropus scapulatus*	2	Skin
	Long tongued nectar bat	*Macroglossus minimus*		
	Dollar bird	*Eurostmus orientalis*	1	Liver
	Indian Koel	*Centropus phasianinus*	1	Liver
	Channel billed cuckoo	*Scythrops novaehollandae*	1	Liver
**Feral vertebrates**			1	Spleen
	European fox	*Vulpes vulpes*	1	Spleen
	Indian mynah	*Acridotheres tristis*	1	Liver
	European starling	*Sturnus vulgaris*	1	Liver
	Feral pig	*Sus scrofa*	20	Diaphragm
	Red deer	*Cervus elaphus*	1	Liver
	Cane toad	*Bufo marinus*	1	Liver
**Marsupials**				
	Common wombat	*Vombatus ursinae*	5	Blood
	Tasmanian devil	*Sarcophilus harisi*	1	DNA
	Red necked wallaby	*Macropus rufogriseus*	4	Spleen
	Brushtail possum	Trichosurus vulpecular*	4	Spleen
	Sugar glider	Petaurus breviceps*	2	Spleen
	Ringtail possum	Pseudocheirus perigrinus*	1	Spleen
	Stripe faced dunnart	*Smithopsis macroura*	5	Liver

Those with an * yielded an amplicon of the appropriate size.

Following positive results in the initial PCR screening of *Melomys spp.* specimens, additional samples from *M. burtonii* and *M. cervinipes* were obtained from other researchers. An additional six *M. burtoni* were trapped as part of this project. Trapping was conducted nocturnally on Bribie Island, between June – August 2008 using Elliot traps.

Traps were set after dark and checked the following morning. Trapped animals were anaesthetized with isofluorane immediately upon removal from the trap and then euthanased by intraperitoneal injection of pentobarbital. Euthanasia by injection of barbiturate was performed in accordance with Standard Operating Procedure AHT 39 as approved by the University of Queensland. Blood was collected by cardiac puncture into 2 ml EDTA tubes (Vacutainer, Becton Dickinson, North Ryde, NSW). Tissue biopsies (approx 0.5 cm^3^) were collected aseptically from liver, kidney and spleen (6 animals) and testis (2 animals) for tissue culture. Including the initial screening, a total of 30 *Melomys spp* samples were tested.

### DNA extraction, PCR and sequencing

DNA was extracted from blood or tissue using either a QIAamp DNA mini kit or a QIAgen DNeasy blood and tissue kit (QIAgen, Hilden, Germany) according to manufacturer’s instructions. Previously published primers were used [6], or were designed using either Vector NTI or Primer 3 programs and based on an alignment of GALV and KoRV sequences, on GALV sequence alone or on sequence from the novel melomys provirus. A total of 22 primer pairs were used either in the initial screening of DNA samples, or to further detect additional KoRV related proviral sequences in the *M.burtoni* DNA. Primers were designed to cover all regions of the MbRV genome from just downstream of the 5’ LTR to just upstream of the 3’LTR. Primers which gave meaningful sequence are listed in Table 2. The remaining primer pairs failed to yield an amplicon.

**Table 2:** PCR primers which yielded partial MbRV proviral sequence.

No	Name	Forward primer	Reverse primer
1*	KoRV Polymerase gene	CCTTGGACCACCAAGAGACTTTTGA	TCAAATCTTGGACTGGCCGA
2	MbRV 2600F-4549R	CCTCTATCGACCCATCCTGG	TAGTTCCTGCCAGCACTCTG
3	MbRV 4015F-5034R	CCAGTGCACAGGTGCTCAG	GCCGGGCCATTGTCTGAC
4	MbRV 6057F –7541R	GTAAAGAWTGGGWTTGTGAGACC	CCTATCATTGATGAATTGWACTAAC

*Reference [6].

The numbers in primer pairs 2,3 and 4 refer to the approximate position in the homologous region of either the KoRV or GALV genome.

### PCR amplification

#### *Polymerase* gene PCR (Pair 1)

The PCR reaction mix comprised 5.0 µl Orange G loading dye, 5.0 µl 10X buffer, 0.2 mM forward primer, 0.2 mM reverse primer, 0.1 µM dNTPs, 3 mM MgCl_2_, 4.0 µl DMSO, approximately 0.2 µg DNA template, 0.25 µl Red Hot Taq (Thermo scientific) and ultrapure water to a final volume of 50.0 µl. Cycling conditions were an initial denaturation of 95°C for 2 minutes, followed by 35 cycles of 95°C for 30 seconds, 50°C for 30 seconds and 72°C for 30 seconds, followed by a final extension of 72°C for 10 minutes.

#### MbRV 2600F-4549R, MbRV 4015F-5034R and MbRV 6057F-7541R PCR (Pairs 2, 3 and 4)

The reaction mix was 0.5 µl iProof long range Taq, 5 µl Orange G, 1 mM MgCl_2_, 0.1 µM dNTPs, 0.2 mM forward primer, 0.2 mM reverse primer, 2.0 µl DMSO, 1.0 µl DNA template and ultra pure water to a final volume of 50.0 µl. Cycling conditions were an initial denaturation at 98°C for 30 seconds, followed by 35 cycles of 98°C for 10 seconds, 50°C for 20 seconds and 72°C for 60 seconds followed by a final extension of 72°C for 10 minutes. When an amplicon of the appropriate size was obtained, the band was excised from the gel and purified using a QIAgen gel purification kit. Sequencing reactions used the BigDye Terminator 3.1 system and DNA sequences were assayed on a ABI/Hitachi 3130xl Genetic Analyzer (Applied Biosystems, Hitachi). Sequences were then screened using the Basic Local Alignment Search Tool (BLAST) [27] in the NCBI database.

### Sequence alignments and phylogenetic analysis

**Figure 1:**
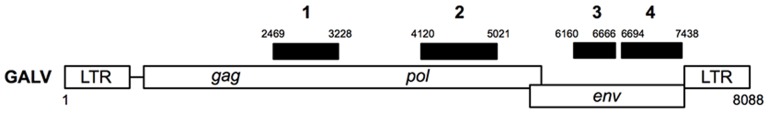
Relative positions of MbRV fragments with respect to GALV genome. Schematic showing the relative positions of the four MbRV fragments (dark bars) and the positions in which they align against the GALV genome. MbRV sequenced fragments are labeled 1–4 starting from the 5′ end the genome.

Four fragments of retroviral sequence were amplified from the DNA (Figure 1) of *M. burtoni* using primers designated in Table 2 providing a total of 2,880 bp sequence. We designated this sequence *Melomys burtoni* retrovirus (MbRV). Sequences 1 and 2 (Genbank KF572483, Genebank KF572484) are from the *pol* gene and sequences 3 and 4 (Genbank KF572485, Genbank KF572486) are from the *env* gene. The MbRV sequences for each concatamer were aligned against all published sequences available from related viruses. The Genbank accession numbers of these sequences is given in Table 3. A strain of GALV isolated from a GALV-SSAV infected marmoset tumour cell line, was designated GALV-MAR. Alignments were performed using the ClustalW program in MEGA 5.1 [28]. The best-fit nucleotide substitution model, determined in jModelTest v2.1.1 [29]–[30], for *env* sequences was general time reversible (GTR) with proportion of invariant sites, I = 0.236 and gamma of 2.738, and for *pol* was HKY [31] with gamma of 0.674. Using these parameters, phylogenetic trees were constructed by Bayesian inference trees in MrBayes v3.2 [32] with 10^6^ generations and a discarded 25% burn-in. The final trees were visualised in FigTree v1.4.0 [33].

**Table 3:** Genebank sequences used in alignments to construct phylogenetic trees.

Gene	Name	Genbank Accession number
*pol, env*	GALV-SEATO	AF055060.1
*pol, env*	GALV-X	U60065.1
*pol, env*	KoRV	AF151794.2
*pol, env*	MDEV	AF053745.1
*pol, env*	FeLV	NC_001940.1
*pol*	MbRV seq 1	KF572483
*pol*	MbRV seq 2	KF572484
*pol*	MlRV	JQ951956.1
*env*	GALV-SF	AF055063.1
*env*	GALV-Br	AF055062.1
*env*	GALV-H	AF055061.1
*env*	SSAV	AF055064.1
*env*	GALV-MAR	U20589.1
*env*	MbRV seq 3	KF572485
*env*	MbRV seq 4	KF572486

### Rodent species identification

Individual rodents were distinguished at the genus level by gross morphology, using features such as the presence of hair and typical patterns on the tail. Distinguishing by gross morphology at the species level in either the *Melomys* or *Rattus* genus requires fine measurement of detailed anatomic characteristics and was not attempted in this study. A tentative assignment of melomys species was made based on the habitat in which the animal was trapped, with those trapped in rainforest habitat considered likely to be *M. cervinipes* and those trapped in dryer schlerophyll forests considered to be *M. burtoni*, although there is an overlap in the range of both species [34]. Definitive species identification of these rodents was conducted using PCR to amplify a 433 bp fragment of the mitochondrial DNA mammalian control region using published primers and protocols [35]. Primers used were Melomys_Spp_F 5′-CTCCACCATCAGCACCCAAAGC-3′ and Melomys_Spp_R 5′-CCTGAAGTAGGAACCAGATG-3′. The PCR amplification was conducted in a 50 µl reaction volume containing 1.25 units of Taq DNA polymerase, 0.1 µM dNTP, 0.2 µM of each primer, 5 µl of 10x PCR buffer and about 0.2 µg of genomic DNA. Cycling conditions were initial denaturation at 94°C for 1 minute, then 30 cycles of 94°C for 30 seconds, 53°C for 30 seconds and 72°C for 1 minute before an extra final extension step at 72°C for 7 minutes. Resulting PCR products were examined on a 0.7% gel, purified by a PCR clean-up kit and sequenced at the AGRF at the University of Queensland, St Lucia.

### Attempted virus isolation and viral RNA detection from melomys specimens

Following euthanasia and collection of blood by cardiac puncture from six melomys trapped on Bribie Island, primary cell cultures were established from a range of tissues including peripheral blood mononuclear cells (PBMCs) in an attempt to isolate a retrovirus. PBMCs were purified using Ficolpaque (Stemcell technologies, Tullamarine, Victoria) either from blood collected into EDTA and diluted 1∶1 in RPMI medium (Life technologies, Mulgrave, Victoria) containing 20% fetal calf serum (RPMI/FCS) or from spleens that had been aseptically removed and flushed with RPMI/FCS. Following purification, the PBMCs were suspended in RPMI/FCS and incubated at 37°C in 5% CO_2_. Some of the PBMC cultures were mitogen-stimulated by the addition of 1% concanavalin A to the culture medium. For other tissue cultures, small (0.5 cm×0.5 cm×0.5 cm) sections of liver, kidney, spleen and the testis were aseptically removed and macerated by grinding over a sterile metal sieve. The macerated tissue was placed into culture dishes, overlaid with DMEM (Life technologies, Mulgrave, Victoria) containing 20% FCS, and incubated as above. Media was replaced approximately every 3 or 4 days and the cultures were observed for cell growth and evidence of viral cytopathic effect. In addition, fresh plasma (200 µl) collected from one of the live specimens trapped on Bribie Island (BRME002) was inoculated into a 25 cm^2^ cell culture flask containing confluent VERO cells. One ml of the supernatant was collected daily for 3 days.

Melomys plasma and samples of culture supernatant from the PBMC and other primary cell cultures were tested by reverse-transcriptase PCR for the presence of viral RNA. RNA was extracted using a QIAgen Viral RNA minikit (QIAgen, Hilden, Germany) and cDNA was produced using a Superscript 111 (Invitrogen, Mulgrave, Victoria) reverse transcription kit according to the manufacturer’s protocol, (except that less than 1 µg RNA was used in the reactions). KoRV viral RNA extracted from koala plasma was used as a positive control in these reactions. PCR using the KoRV *polymerase* gene primers was then performed.

### Electron microscopy

Cell pellets from the mitogen-stimulated PBMC cultures were examined by electron microscopy. Briefly, ultra-thin sections (60 nm) of glutaraldehyde-fixed pelleted cells that had been processed using standard methods were placed on coated grids. Transmission electron microscopy was performed using a Jeol 1010 Transmission Electron Microscope (Jeol Ltd. Japan).

## Results

### PCR screening

The PCR primer pair used in the screening that gave the most consistent results in terms of producing clear amplicons of appropriate size was the KoRV *polymerase* gene pair.

Of the DNA extracted from 42 species and screened using these primers, eight species yielded amplicons of the expected size (summarised in Table 1). These species were *Mus musculus*, *Uromys caudimaculatus, Rattus sordidus*, *Pteropus alecto, Trichosurus vulpecular, Pseudocheirus pererinus, Petaurus breviceps and Melomys burtoni*.

All 30 samples from *Mus musculus* gave an amplicon of the same size, of which 15 were sequenced and had highest identity with *Mus musculus* genomic sequence from chromosome 7 (Acc No151412.2). Two of three samples of white-tailed rat (*Uromys caudimaculatus*) provided sequences that were similar to *Mus musculus*, chromosome 18 (AccNo124717.3). One of three canefield rat (*Rattus sordidus*) samples gave a sequence similar to *Felis catus*, chromosome unknown (AccNo235681.1). All of these sequence matches most likely represent homologies with species specific endogenous retroviral elements.

All black flying fox (*Pteropus alecto*) samples yielded appropriately sized amplicons (∼400 bp). However, although all these sequences were very similar to each other, the sequences were not recognized as being similar to any known sequence when subjected to a BLAST analysis. Similarly, samples from brushtail possums (*Trichosurus vulpecula*), a ringtailed possum (*Pseudocheirus pererinus*) and sugar gliders (*Petaurus breviceps*) yielded clear amplicons, whose nucleotide sequences were not similar to any sequence on the NCBI database.

In the initial screening, five specimens from *Melomys burtoni* were assayed. All five yielded amplicons of the appropriate size. These samples included skin, spleen, heart and skeletal muscle. Following these findings, additional specimens of *M. burtoni* and *M cervinipes* were tested.

### Melomys retrovirus

Of a total of 30 *Melomys spp* samples tested, 17 were from *Melomys burtoni* and 13 were from *M. cervinipes*. None of the 13 *M. cervinipes* samples yielded an amplicon using the KoRV *polymerase* gene primers. However, all 17 *M. burtoni* were positive using these primers. The nucleotide sequence of this amplicon revealed a close similarity with sequences from both KoRV and GALV. We designated this proviral sequence Melomys burtoni retrovirus (MbRV).

The sequence from this amplicon (approximately 400 bp) had 100% identity among all *Melomys burtoni* specimens tested. Primer pairs 2, 3 and 4 were used to amplify additional proviral fragments, which also yielded sequence with close identity to KoRV and GALV. In total, 2880 bp of MbRV sequence was obtained.

Attempts to isolate additional sequences of MbRV and to characterize the full genome are ongoing. However phylogenetic trees for the individual sequences (data not shown) place the 4 fragments in similar positions within the phylogeny when compared to the phylogenies of the concatenated sequences suggesting that these sequences are from the same provirus. It remains possible however that one or more of these fragments are from different proviruses.

The 4 MbRV amplicons had 94%, 93%, 92% and 90% nucleotide identity with GALV-SEATO and 84%, 82%, 74% and 79% identity with KoRV respectively.

**Figure 2:**
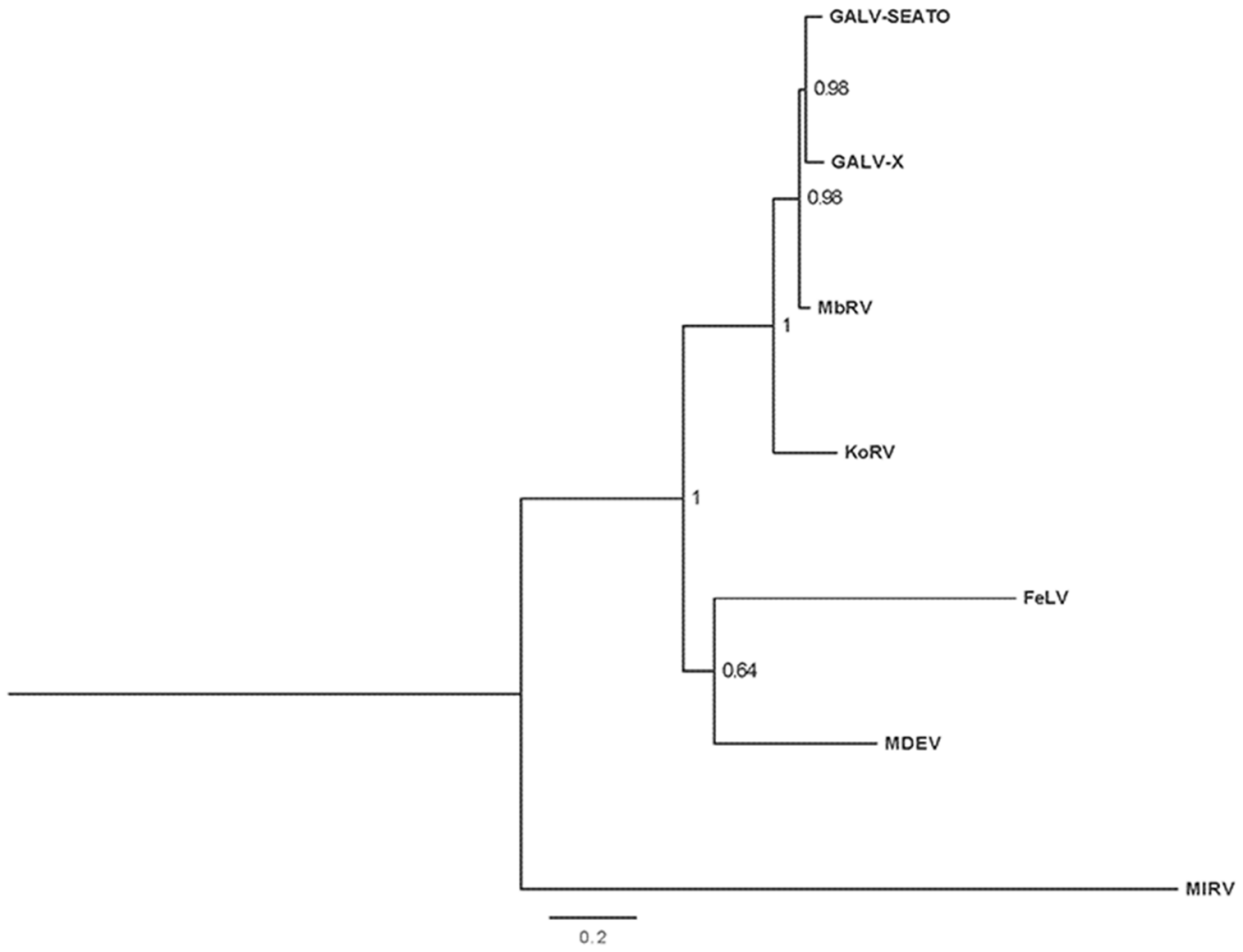
Phylogenetic tree for concatenated *pol* sequences. Bayesian inference tree for the concatenated *pol* sequences of MbRV and related sequences available on Genbank. Numbers given at nodes represent Bayesian posterior probabilities; scale bar represents 0.2 substitutions per site. The tree was midpoint rooted. Taxa abbreviations are described in Table 3.

**Figure 3:**
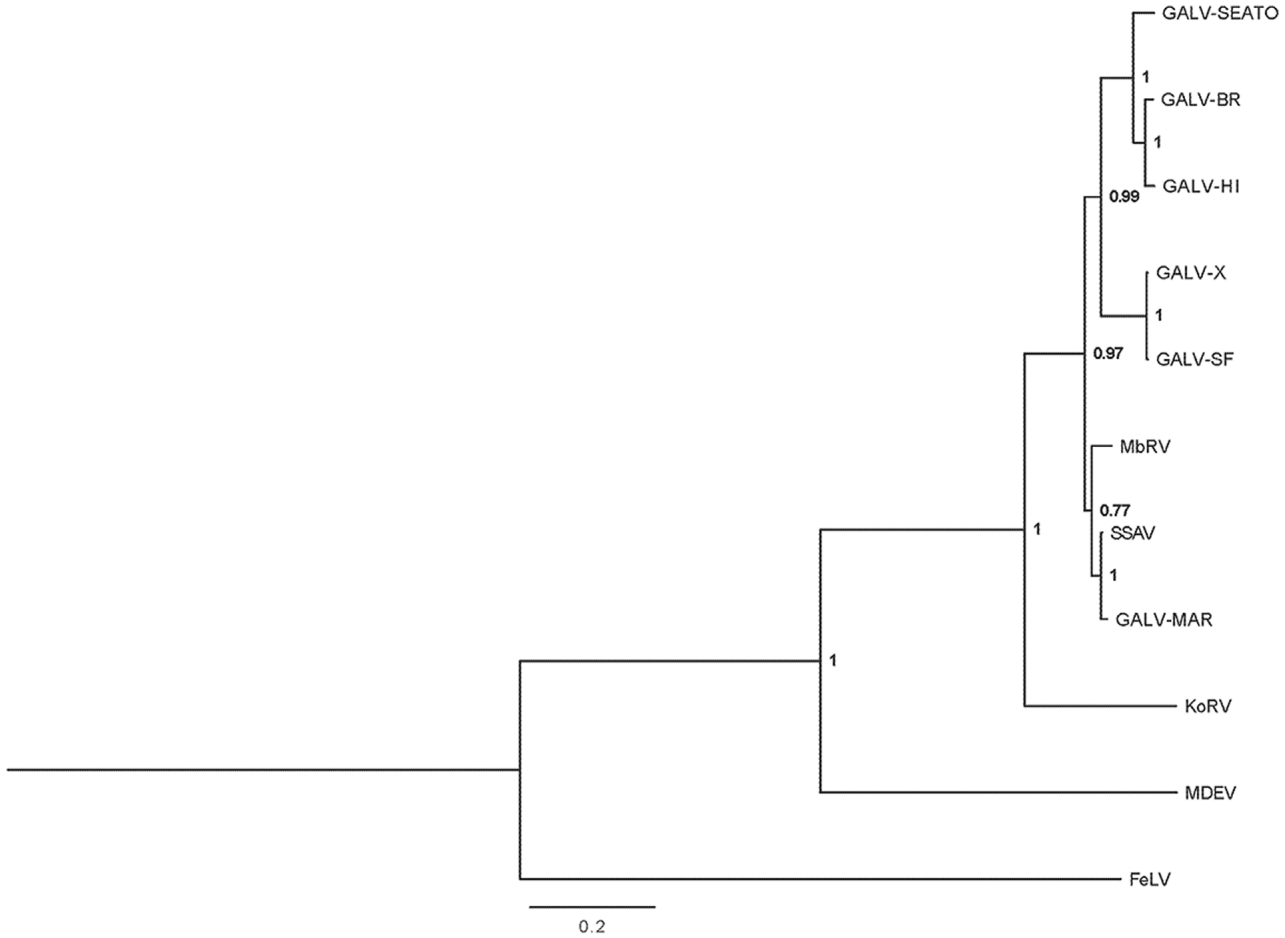
Phylogenetic tree for concatenated *env* sequences. Bayesian inference tree for the concatenated *env* sequences of MbRV and related sequences available on Genbank. Numbers at nodes represent Bayesian posterior probabilities; scale bar represents 0.2 substitutions per site. The tree was midpoint rooted. Abbreviations described in Table 3.

The concatenated sequence of MbRV amplicons showed 93% nucleotide identity with GALV-SEATO and 83% identity with KoRV. In phylogenetic analysis, MbRV formed close relationships with the GALV sequences; for the *pol* gene, MbRV was placed as a sister taxon to two GALV sequences (Figure 2) and for the *env* gene, GALV and MbRV sequences formed a monophyletic clade (Figure 3). These relationships showed high posterior probability support. In both trees, MbRV, GALV and KoRV sequences formed a well-supported monophyletic clade to the exclusion of feline leukemia virus (FeLV) and Mus dunni endogenous virus (MDEV).

### Attempted virus isolation and viral RNA detection

PBMCs and fibroblast cells from both *M. burtoni* spleen and testis were successfully cultured from six animals, with cells surviving for up to 20 days. No viral cytopathic effect was observed in any of the cell cultures. All attempts to detect MbRV viral RNA directly from plasma, from Con A-stimulated PBMC supernatants, from Vero cell cultures which had been inoculated with plasma or from cultured fibroblasts derived from spleen were unsuccessful.

### Transmission electron microscopy

Approximately 20 sections of Con A-stimulated PBMC cultures were examined by electron microscopy. Although a number of structures of an appropriate retrovirus size were visualized, nothing with typical gammaretrovirus morphology was observed.

## Discussion

The nucleotide sequences of the four MbRV fragments derived from DNA samples from *Melomys burtoni* are remarkably similar to sequences of GALV and, to a lesser extent, of KoRV. This is reinforced by the Bayesian inference trees for the *env* fragments, in particular, which cluster MbRV and GALV sequences in a monophyletic clade. Based on this relationship, MbRV may be considered a sub-type of GALV. When the degree of similarity between KoRV and GALV became known it stimulated much interest in the origins of both viruses [24]. The discovery of these MbRV sequences provides an additional and intriguing perspective.

Based on the data presented here it seems likely that MbRV is an endogenous virus. Proviral sequence was present in 100% of *M. burtoni* specimens tested and attempts to demonstrate the presence of viral RNA either directly in plasma or in PBMC or other primary cell cultures were unsuccessful. In addition, electron microscopy failed to demonstrate typical gammaretrovirus type particles in mitogen-stimulated PBMC cultures. However, these attempts to isolate virus were not exhaustive and it is still possible that MbRV could be transcriptionally active in different culture systems. In support of this possibility, the MbRV sequences detailed here all contained homologous open reading frames, which is consistent with either an exogenous or recently endogenised virus.

A greater understanding of the biology of the melomys host may help to shed light on the close genetic relationship between KoRV, GALV and MbRV. Melomys are murine rodents that are thought to have arrived in Australia about 5 million years ago via the land bridge with Papua New Guinea [36]. Of the five or six melomys species in Australia, [37], the grassland melomys (*M. burtoni*) and the fawn footed melomys (*M. cervinipes*) are the most abundant. Both of these species are found in coastal regions of north eastern Australia with *M. cervinipes* found in wetter rainforest habitats and *M. burtoni* found in drier grassland habitats, although their distribution overlaps in intermediate habitats [34].

Although both melomys species examined in this study are closely related and in some regions share a common habitat, there was no evidence of MbRV sequence in any of the *M. cervinipes* tested while all *M. burtoni* tested were positive. It is possible that despite their close relatedness there is sufficient genetic variation to make *M. cervinipes* resistant to MbRV infection, for example through variation in the cell receptor for the virus. The murine APOBEC3 gene has been shown to restrict infection with Moloney murine leukaemia virus in mice, with mice lacking functional copies of this gene being more susceptible to infection [38]. Thus it may be that *M. cervinipes*, but not *M. burtoni*, has evolved restriction factors which render it resistant to infection with MbRV. Alternatively, it is possible that lack of physical contact between *M. cervinipes* and *M. burtoni* individuals, even in areas where their geographical distribution overlaps, prevented viral transmission between the two species. *M. cervinipes* is arboreal while *M. burtoni* spends a greater part of its time on the ground, so perhaps close interactions between the two species are uncommon. This, combined with the possibility that *M. burtoni* individuals may rarely be viraemic, could explain the lack of MbRV in *M. cervinipes*. It is also possible that *M. cervinipes* do carry MbRV-related sequences but at a lower copy number or at a lower prevalence in the population than could be detected in this study.

Considering the geographic distribution and the phylogenetic placement of melomys, koalas and gibbons, there is no clear explanation for the close genetic relationships between MbRV, KoRV and GALV. The sequence integrity, heterogeneity, instability and clinical associations of KoRV and the clinical pattern of GALV infection are consistent with naïve host events, suggesting cross-species virus transmission events. Although MbRV is a potential ancestor virus, geographic and biological obstacles make it somewhat difficult to create plausible scenarios to explain such transmission events between melomys and the other two host species, particularly gibbons.

Although grassland melomys and koalas share a similar geographic ranges down the east coast of Australia [1], [34] and both species are nocturnal, koalas are mostly arboreal, whereas grassland melomys are terrestrial. However, koalas spend short periods on the ground, particularly during the breeding season [1] and it is therefore possible that individuals of the two species do occasionally interact, allowing viral transmission between these species. However, considering that *M. burtoni* and koalas have likely been present in Australia for 5 million years and 15 million years, respectively [1], [36], it is perhaps surprising that KoRV has putatively only been present in koalas for about 200 years [24]. It is possible either that suitable interactions between koalas and viraemic melomys are very rare events or that the calculations are incorrect. Recent studies on archival koala samples have suggested a longer association between this retrovirus and its koala host than previously thought [39]. A longer time frame in which the putative cross species transmission of MbRV to koalas occurred would allow for some genetic divergence between MbRV and KoRV to occur which would explain why KoRV and MbRV do not share a higher degree of similarity today. In addition it is possible that the initial putative cross species transmission of MbRV to koalas and the subsequent endogenization of KoRV are separated by a considerable period of time.

In contrast to the potential interactions between koalas and melomys, it is very difficult to explain a connection between melomys and gibbons and to understand the very close genetic relatedness of MbRV and GALV. The genus *Melomys* is only found east of Wallace’s Line (between the Indonesian islands of Bali and Lombok) and is primarily limited to Australia, Papua New Guinea and the western Pacific. *Melomys spp* do not occur in mainland South East Asia [40], where GALV emerged. Thus it seems extremely unlikely that there has been natural transmission of virus between melomys and gibbons in Thailand. It is possible that there was some form of iatrogenic transmission of MbRV or a MbRV-like virus to gibbons in the Bangkok colony. It is also possible that another as yet unknown host, which is distributed on both sides of Wallace’s Line and which harbours a virus similar to GALV or MbRV, could have introduced the virus to the gibbon colony through natural contact. Whether this unknown host was a rodent or another mammal and whether it was a native of Thailand, a long-standing feral animal or a recent introduction can only be speculated. Currently there are no published reports of a retrovirus in any other species with the same degree of homology that MbRV shares with KoRV or GALV. Several early reports suggested the presence of GALV-related virus in some rodents, but these experiments were based on DNA hybridization techniques which were relatively crude in determining sequence homology [41]–[42]. Thus despite our detection of a closely related virus sequence in an Australian native rodent, the origins of GALV remain obscure.

Clearly, further research is required to better understand the relationships between MbRV, KoRV and GALV. Additional sequencing and culture attempts may reveal whether full-length MbRV provirus is present and transcriptionally active. Investigation of a larger number of *M. burtoni* specimens will confirm or contradict the finding of 100% prevalence we report here, and testing of other melomys species will help to clarify the host specificity of this virus. Such studies would help to elucidate whether MbRV is an endogenous virus and may help to unravel the mystery surrounding this group of retroviruses.
